# ﻿DNA barcoding of *Scomberomorus* (Scombridae, Actinopterygii) reveals cryptic diversity and misidentifications

**DOI:** 10.3897/zookeys.1135.93631

**Published:** 2022-12-14

**Authors:** Xiao-Shu Zeng, Cheng-He Sun, Xiao-Ying Huang, Ye-Ling Lao, Jin-Long Huang, Sha Li, Qun Zhang

**Affiliations:** 1 Department of Ecology and Institute of Hydrobiology, Jinan University, Guangzhou 510632, China Jinan University Guangzhou China; 2 Chinese Sturgeon Research Institute, China Three Gorges Corporation, Yichang 443100, Hubei, China Chinese Sturgeon Research Institute, China Three Gorges Corporation Yichang China; 3 Hubei Key Laboratory of Three Gorges Project for Conservation of Fishes, Yichang 443100, Hubei, China Hubei Key Laboratory of Three Gorges Project for Conservation of Fishes Yichang China

**Keywords:** COI, conservation, cryptic species, DNA-based species delimitation, mackerel, phylogeny

## Abstract

The genus *Scomberomorus* is economically important; however, the taxonomic status and phylogenetic relationships in this genus are not clearly resolved, making it difficult to effectively protect and exploit fish resources. To clarify the taxonomic status of *Scomberomorus* species, mitochondrial cytochrome c oxidase I (COI) gene sequences of 150 samples were analyzed. The average genetic distance among 14 species was approximately 11 times greater than the distances within species, in accordance with the ‘10× rule’ of species identification. Five of the 14 species did not form monophyletic clades based on a Bayesian inference gene tree. The application of four DNA-based species delimitation methods (automatic barcode gap discovery, barcode index numbers, Poisson tree process, and the K/θ method) yielded several key results. (1) Cryptic species were detected within *Scomberomoruscommerson*. (2) A *Scomberomorusqueenslandicus* sample from Australia was misidentified as *S.commerson* in the Barcode of Life Data System (BOLD). (3) Specimens originally identified as *Scomberomorusguttatus* was differentiated into four OTUs or species, two in the Yellow, South China, and Java seas, and two in geographically distant areas, one each in the Arabian Sea and the Bay of Bengal. (4) Six specimens from South Africa originally identified as *S.plurilineatus* most likely do not belong to the species. (5) Specimens identified as *S.maculatus* and *S.regalis* were conspecific; however, introgression cannot be ruled out. Our findings revealed cryptic diversity and difficulties in morphological identification of species in the genus *Scomberomorus*. This study provides scientifically based support for the conservation of germplasm resources of the genus *Scomberomorus*.

## ﻿Introduction

DNA barcoding provides a complementary approach to morphological species identification (Hebert et al. 2003a). The approximately 650 bp sequence at the 5’ end of the animal cytochrome c oxidase I (COI) is a standard DNA barcoding region for delineating species (Hebert et al. 2003a; Vences et al. 2005). Hebert et al. (2003b, 2004) proposed the ‘10× rule’ of species identification and concluded that intraspecific COI genetic distances are generally less than 2% based on an analysis of 13320 species in 11 phyla. Numerous studies (e.g., Zemlak et al. 2009; Pereira et al. 2013; Knebelsberger et al. 2015; Neves et al. 2020) have suggested that the COI gene is effective for differentiating among fish species and could be used for resolving synonymy, heteronomy, and identifying cryptic species.

The genus *Scomberomorus* belongs to the family Scombridae, one of the most popular and familiar food fish in the world (Yemmen and Gargouri 2022), composed of 18 species (Collette et al. 2001). They are rich in protein and highly unsaturated fatty acids and therefore possess high nutritional value (Lou et al. 2000). The biological characteristics and trends in the unit production of *Scomberomorus* fishes indicate that their resources are in a state of decline, and this can be attributed to overfishing and marine environmental pollution (Zheng et al. 2014). Therefore, research focused on the conservation of fish germplasm resources in the genus *Scomberomorus* is urgently needed.

Species identification in the genus *Scomberomorus* is mostly based on morphology (Collette and Russo 1985; Collette et al. 2001; Zhang et al. 2013a) and molecular data (e.g., Hoolihan et al. 2006; Habib and Sulaiman 2016; Mansourkiaei et al. 2016). Morphological identification mainly depends on the lateral line, pattern, and body color (Collette and Nauen 1983). However, the phenotype changes with growth, the color fades during preservation, and sexual dimorphism has been discovered (Collette and Nauen 1983) in some species of this genus, all of which make morphological identification difficult.

Previous molecular studies of the genus *Scomberomorus* have mostly focused on a few species within the genus (e.g., Habib and Sulaiman 2016; Mansourkiaei et al. 2016), and few studies (Banford et al. 1999; Jeena et al. 2022) have evaluated the whole genus. Owing to this lack of species representation, relationships within the *Scomberomorus* are unclear (Bayona-Vásquez et al. 2018). In this study, we conducted a DNA barcoding study of 150 specimens from the genus *Scomberomorus* to clarify their identification and provide scientific support for the conservation of germplasm resources.

## ﻿Materials and methods

### ﻿Ethical statement

The collection and sampling of specimens were reviewed and approved by the Animal Ethics Committee of Jinan University. All specimens used in this study were collected in accordance with Chinese laws. All experiments were performed to ensure optimal animal welfare and care.

### ﻿Sample collection and morphological identification

**Figure 1.**
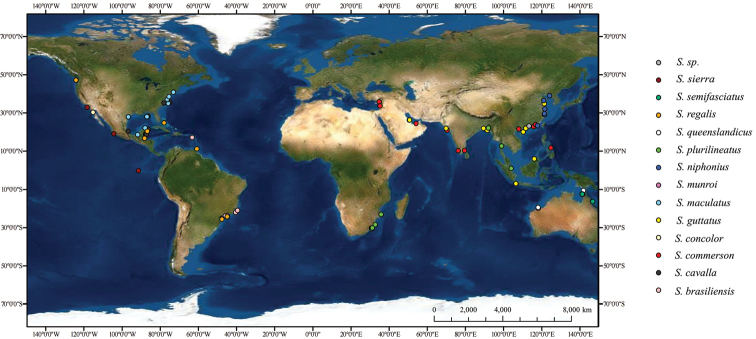
Localities of 150 samples in this study. One dot may represent more than one specimen.

Samples were collected from 11 locations in the coastal waters of China and 116 homologous sequences were downloaded from GenBank and the Barcode of Life Data System (BOLD). The collected information is shown in Fig. 1 and Suppl. material 1: table S1. All specimens preserved in 95% ethanol in Department of Ecology and Institute of Hydrobiology, Jinan University were identified based on morphological characters known in literature (Institute of Zoology et al. 1962; Collette and Russo 1985). For 116 homologous sequences downloaded from GenBank and BOLD, samples were morphologically identified according to the original publications (Suppl. material 1: table S1), excluding directly submitted sequences.

### ﻿DNA extraction, amplification, and sequencing

Muscle tissue samples collected in our laboratory were extracted for the determination of COI sequences. DNA was extracted using a modified phenol/chloroform method (Le et al. 2010). PCR amplification was performed according to the method described by Ward et al. (2005). Universal primers FishF1 (5′-TCA ACC AAC CAC AAA GAC ATT GGC AC-3’) and FishR1 (5′-TAG ACT TCT GGG TGG CCA AAG AAT CA-3’) were used. The 20 μL polymerase chain reaction (PCR) mixtures contained 7 μL of sterilized ultrapure water, 10 μL of PCR Mix, 1 μL of each primer, and 1 μL of the DNA template. The reaction conditions were as follows: pre-denaturation at 95 °C for 5 min, denaturation at 94 °C for 40 s, annealing at 54 °C for 40 s, extension at 72 °C for 50 s, for 35 cycles, and a final extension at 72 °C for 10 min. PCR products were detected by 1% agarose gel electrophoresis, purified, and sequenced by BGI Genomics Co., Ltd. (Shenzhen, China). DNA extraction, amplification, and sequencing of the downloaded homologous sequences were also performed according to GenBank and BOLD.

### ﻿Data analyses

The sequencing peaks were visualized using Chromas 2.6.6 (Technelysium Pty Ltd., South Brisbane, Queensland, Australia 2018) and the sequences were manually calibrated using Bioedit 7.2.5 (Hall 1999). Sequence characteristics were analyzed using MEGA 7.0 (Kumar et al. 2016), and various indices, such as base composition, variable sites (including parsimony-informative sites and singleton sites), and the transition-to-transversion ratio, were calculated.

All genetic distances were calculated based on Kimura two parameter (K2P) (Nei and Kumar 2000) distances using MEGA 7.0 (Kumar et al. 2016). A comparative analysis of all individuals within the same species was used to calculate the genetic distances between samples within each species, and these results were combined with the interspecific genetic distances to plot the barcode gap map of 14 species.

The COI gene sequences were tested for saturation using DAMBE 7.3.5 (Xia and Xie 2001). Based on the K2P substitution model, a neighbor-joining tree (NJ tree) was constructed, branch support was evaluated by 1000 repetitions of sampling, and genetic distances between and within clades were calculated. The construction of the Bayesian inference gene tree (BI tree) was performed using PhyloSuite v. 1.2.2 (Zhang et al. 2020). ModelFinder was used to select the best-fit partitioning model using the BIC criterion (Kalyaanamoorthy et al. 2017). BI phylogenies were inferred using MrBayes 3.2.6 (Ronquist et al. 2012) under the HKY+I+G+F model (two parallel runs, 2000000 generations), in which the initial 25% of sampled data were discarded as burn-in. FigTree v. 1.4.4 (Rambaut 2009) was used to visualize and edit the BI tree.

We employed four species delimitation methods: (1) Automatic Barcode Gap Discovery (ABGD) (Puillandre et al. 2012); (2) Barcode Index Numbers (BIN) (Ratnasingham and Hebert 2013) implemented in BOLD to obtain operational taxonomic units (OTUs); (3) Poisson tree processes (PTP) (Zhang et al. 2013b) implemented in the bPTP server (https://species.h-its.org/ptp/) with the BI tree as the input file; (4) the K/θ method: for species morphologically identified as conspecific, the mean pairwise distance within each clade (θ) and the minimum pairwise distance between clades (K) in the phylogenetic tree were recorded. Clades with K/θ ≥ 4 are considered reciprocally monophyletic with ≥ 95% probability (Birky et al. 2010).

## ﻿Results

### ﻿Sequence analysis

The COI gene sequences had an average length of 648 bp (567–652 bp) in 150 samples from 14 species in the genus *Scomberomorus*, and there were no base insertions or deletions. There were 453 conserved bases, accounting for 69.48% of the total number of bases, and 199 variable bases, accounting for 30.52% of the total number of bases, including 191 parsimony-informative sites and eight singleton bases. The A+T content (53.3%) was higher than the C+G content (46.7%), indicating an AT bias. The transition-to-transversion ratio was 3.1. A saturation analysis (Suppl. material 1: table S2) indicated that the base mutations did not reach saturation and were suitable for phylogenetic analyses.

### ﻿Genetic distances and barcoding gaps

**Figure 2.**
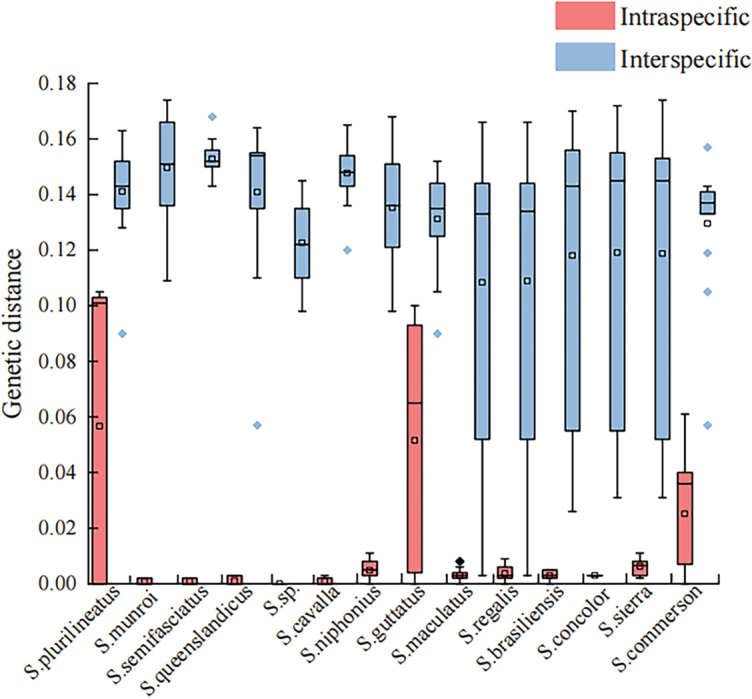
DNA barcoding gaps of 14 *Scomberomorus* species

The intraspecific genetic distances of 14 species of the genus *Scomberomorus* were 0%–6.0%, with an average genetic distance of 1.18%. The interspecific genetic distances were 0.3%–17.4%, with an average genetic distance of 13.0%, which was approximately 11 times higher than estimates within species. Furthermore, 79% of intraspecific genetic distances were within the range of 0%–2%. The barcoding gap map (Fig. 2) showed that the maximum intraspecific genetic distances for *S.commerson* (Lacepède, 1800), *S.guttatus* (Bloch & Schneider, 1801), *S.plurilineatus* Fourmanoir, 1966, *S.regalis* (Bloch, 1793), and *S.maculatus* (Mitchill, 1815) were not clearly different from the minimum interspecific genetic distances. In particular, the intraspecific genetic distances for *S.plurilineatus*, *S.guttatus*, and *S.commerson* were 6%, 5.2%, and 2.6%, respectively, and the interspecific genetic distance between *S.maculatus* and *S.regalis* was 0.3%. The remaining nine species had intraspecific distances of less than 2% and interspecific distances greater than 2%, forming clear DNA barcoding gaps.

### ﻿Phylogenetic clustering analysis

**Figure 3.**
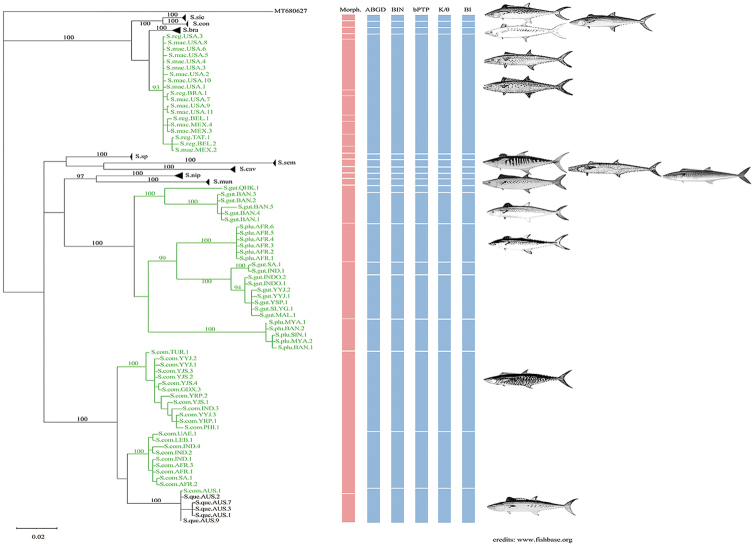
Bayesian inference (BI) tree based on the COI sequences of 14 *Scomberomorus* species. The green clades represent five species for which the species delimitation result is different based on morphology and the BI tree: *S.commerson*, *S.guttatus*, *S.plurilineatus*, *S.regalis*, and *S.maculatus*. Images of the genus *Scomberomorus* on the right from top to bottom are: *S.sierra*, *S.concolor*, *S.brasiliensis*, *S.regalis*, *S.maculatus*, *S.semifasciatus*, *S.cavalla*, *S.niphonius*, *S.munroi*, *S.guttatus*, *S.plurilineatus*, *S.commerson*, and *S.queenslandicus*. MT680627 is the outgroup. Numbers near the branches are bootstrap values.

A BI tree (Fig. 3) and a NJ tree (Suppl. material 1: fig. S1) exhibited similar topologies. All specimens in the BI tree formed 18 clades. The average genetic distance between clades was 12.97% (2.3%–17.4%), which was 48 times higher than the average genetic distance within clades of 0.27% (0%–0.75%). *S.commerson* formed two clades with 100 bootstrap values separated by a genetic distance of 3.9% in the BI tree. One *S.commerson* sample from Australia was assigned to a lineage with *S.plurilineatus* samples from Australia. *S.plurilineatus* and *S.guttatus* clustered together in one large clade, and *S.guttatus* formed three clades with 100, 100, and 94 bootstrap values in the BI tree. Together with the sample from Haikou, Hainan, China, *S.guttatus* was divided into four small clades with an inter-clade genetic distance of 7.48%, which was 31 times higher than the average intra-clade genetic distance (0.24%). *S.maculatus* formed a clade with *S.regalis* with 93 bootstrap value in the BI tree, and the genetic distance within the clade was only 0.3%. All other species formed monophyletic groups.

### ﻿DNA-based species delimitation

Four DNA-based species delimitation methods yielded consistent results (Fig. 3). All methods supported the division of 14 species into 18 hypothetical species by classifying *S.commerson* into two hypothetical species, *S.guttatus* into four hypothetical species, and *S.plurilineatus* into two hypothetical species and combining *S.regalis* with *S.maculatus*.

**Figure 4.**
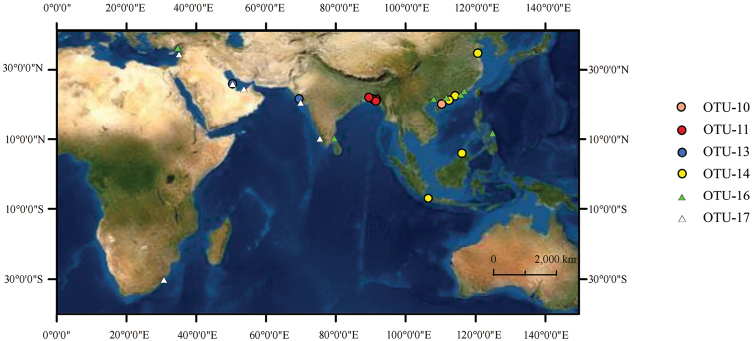
Localities of new clades of *S.guttatus* (OTU-10, OTU-11, OTU-13, and OTU-14) and *S.commerson* (OTU-16 and OTU-17) based on the bPTP results.

In the ABGD analysis (Suppl. material 1: fig. S2), a good barcode gap was observed when a priori intraspecific divergence was 0.0046, and this barcode gap strongly supported the division of taxa into 18 groups. The BIN analysis (Suppl. material 1: table S3) divided the 150 samples into 18 OTUs in which the genetic distance between the OTU and the nearest OTU (NN Dist) was greater than the internal maximum genetic distance (Max), indicating apparent divergence. In the bPTP analysis (Table 1), combined with the results based on morphological characters, the divisions based on bPTP (ML) were selected as the species definitions. A map (Fig. 4) of new clades of *S.guttatus* (OTU-10, OTU-11, OTU-13, and OTU-14) and *S.commerson* (OTU-16 and OTU-17) was obtained based on the bPTP results. In the K/θ method (Suppl. material 1: table S4), the clades with θ = 0 and the clade with only one sample (clade 10 in the BI tree) were considered separate OTUs; therefore, 18 OTUs were obtained.

**Table 1.** Results for 14 *Scomberomorus* species based on a bPTP analysis.

Morphological species	OTU	Number	Catalog number
*sierra*	OTU-1	4	S.sie.ECU.1, S.sie.MEX.1–2, S.sie.USA.1
*S.concolor*	OTU-2	2	S.con.MEX.1, S.con.MEX.2
*S.brasiliensis*	OTU-3	14	S.bra.NET.1, S.bra.BRA.1–13
*S.maculatus* and *S.regalis*	OTU-4	28	S.mac.USA.1–11, S.mac.MEX.1–5, S.reg.BRA.1–3, S.reg.BEL.1–3, S.reg.TAT.1, S.reg.BAH.1, S.reg.MEX.1, S.reg.USA.1–3
*S. sp*	OTU-5	3	S.sp.YSP.1–3
*S.semifasciatus*	OTU-6	5	S.sem.AUS.1–5
*S.cavalla*	OTU-7	12	S.cav.USA.1–2, S.cav.MEX.1–7, S.cav.ALT.1, S.cav.USA.3–4
*S.niphonius*	OTU-8	14	S.nip.SLS.1–4, S.nip.LYK.1–3, S.nip.YRP.1–2, S.nip.LDG.1–3, S.nip.ZSM.1, S.nip.ZZS.1
*S.munroi*	OTU-9	6	S.mun.AUS.1–6
*S.guttatus*	OTU-10	1	S.gut.QHK.1
	OTU-11	5	S.gut.BAN.1–5
	OTU-12	6	S.plu.AFR.1–6
	OTU-13	2	S.gut.IND.1, S.gut.SA.1
*S.plurilineatus*	OTU-14	9	S.gut.INDO.1–2, S.gut.MAL.1, S.gut.SCS.1, S.gut.SLYG.1, S.gut.YSP.1–2, S.gut.YYJ.1–2
	OTU-15	5	S.plu.MYA.1–2, S.plu.BAN.1–2, S.plu.SIN.1
*S.commerson*	OTU-16	15	S.com.IND.3, S.com.PHI.1, S.com.GDX.1–3, S.com.YYJ.1–3, S.com.YRP.1–2, S.com.YJS.1–4, S.com.TUR.1
	OTU-17	9	S.com.AFR.1–3, S.com.SA.1, S.com.IND.1–2, S.com.IND.4, S.com.LEB.1, S.com.UAE.1
*S.queenslandicus*	OTU-18	10	S.com.AUS.1, S.que.AUS.1–9

## ﻿Discussion

According to the ‘10× rule’ of species identification and 2% threshold (Hebert et al. 2003b, 2004), taxonomic uncertainty was discovered in the samples of *S.commerson*, *S.guttatus*, *S.plurilineatus*, *S.maculatus*, and *S.regalis* on the basis of genetic distances. Phylogenetic trees and four DNA molecular definition methods all support the classification of 14 morphological species into 18 hypothetical species.

### ﻿Taxonomic identification of *S.commerson* and *S.queenslandicus* individuals

Intraspecific genetic distance in *S.commerson* (2.6%) was slightly greater than the threshold of 2%; *S.commerson* samples were assigned to three lineages in the phylogenetic trees. The samples from Australia were mixed with *S.queenslandicus* (Munro, 1943) on a single clade. Both species are distributed in Australia (Collette and Nauen 1983; Collette et al. 2001; Kuiter 2021). The adult color pattern in the two species differ: *S.commerson* has many thin, wavy, vertical stripes on the side of the body, while *S.queenslandicus* adults have about three indistinct rows of bronze-grey blotches on the sides (Collette and Nauen 1983). Adult *S.commerson* (generally 120 cm in length) is larger than *S.queenslandicus* (generally 80 cm in length), and juvenile *S.commerson* often have blotches. Therefore, we assumed that *S.commerson* from Australia in the BOLD was misidentified and was actually *S.queenslandicus*; however, further identification and determination were impossible because the corresponding image was not available in the database.

The bootstrap values for the other two clades of *S.commerson* were 100 in the BI tree. The samples in this study and the three samples from India, the Philippines, and Turkey were assigned to the same clade, and the remaining samples belonged to a separate clade. The two clades did not show obvious geographical clustering (Fig. 4), and the genetic distance between clades was 3.9% (greater than the 2% threshold). Vineesh et al. (2018) detected at least three genetically distinct populations of *S.commerson* in the Indian Ocean region. Johnson et al. (2021) studied the genetic population structure and phylogenetic relationships in the coastal waters of northern Tanzania and concluded that *S.commerson* is a single mixed population with high genetic diversity. In this study, the splitting of *S.commerson* into two clades in the molecular phylogenetic trees without obvious geographical clustering may be explained by the migratory behavior of the mackerels and the easy dispersal of their larvae over a wide area by ocean currents (Hoolihan et al. 2006), leading to secondary contact after differentiation. Therefore, cryptic species may exist in this lineage.

### ﻿Taxonomic identification of *S.guttatus* and *S.plurilineatus* individuals

The intraspecific genetic distance in *S.guttatus* was 5.2%, which was significantly greater than the 2% threshold. Four clades were formed in the phylogenetic trees, and the genetic distances between the clades were greater than 2% in the phylogenetic trees. The inter-clade genetic distance (7.48%) was 31 times higher than the average intra-clade genetic distance (0.24%), which was in accordance with the ‘10× rule’ of species identification (Hebert et al. 2003b, 2004). Except for the sample from Haikou, Hainan, China, the other three clades clustered according to geographical distributions. The first clade consisted of individuals from the Bay of Bengal, the second clade consisted of individuals from the Arabian Gulf and Arabian Sea, and the third clade consisted of individuals from the Yellow Sea, South China Sea, and Java Sea (Fig. 4). Yu et al. (2021) conducted a DNA barcoding analysis of *Jaydiasmithi* (Kotthaus, 1970) and found that the Chinese and Mediterranean populations could be divided into two groups, with an average genetic distance between the two groups of 0.044, suggesting the presence of cryptic species. Chen et al. (2017) found that the genetic distance between two *Teraponputa* (Cuvier, 1829) groups was 5% in the western Pacific and Indo-Mediterranean and suggested that the species might be divided into two subspecies or even two species. Luo et al. (2021) found that the average intraspecific genetic distance of *Lateolabrax* spp. was 3.91%, and two clades corresponding to populations in China and Japan were found in the NJ tree; the average inter-clade genetic distance (6.98%) was 14.2 times higher than the average intra-clade genetic distance (0.49%), supporting the division into two species, *L.japonicus* and *L.maculatus*. A study of *S.guttatus* from the South China Sea (Ye 2012) revealed that 19 individuals formed two major clades and suggested that the group originated from two different maternal ancestors. These findings are highly similar to the results of the present study, in which samples from the South China Sea clustered into two clades. Our data indicate that *S.guttatus* complex involves four OTUs or even four species.

*S.plurilineatus* formed two distant clades in the phylogenetic trees, one of which mixed with *S.guttatus*. the name *S.guttatus* has been misapplied to *S.plurilineatus* (Collette and Nauen 1983); therefore, six specimens of *S.plurilineatus* from South Africa might have been misidentified. According to a previous publication (Steinke et al. 2016), three specimens (JF494458–JF494460) were adults and morphological identification was performed; however, specimen images were not available in GenBank or BOLD. The other three specimens (DSLAG600-10, DSLAG1283-11, and DSLAG1287-11) were larvae and images of juvenile and fish eggs were obtained; however, it was not possible to accurately identify them based on the images alone.

### ﻿Taxonomic identification of *S.maculatus* and *S.regalis* individuals

The interspecific genetic distance between *S.maculatus* and *S.regalis* was 0.3% and the taxa were mixed on a single clade of the phylogenetic trees. According to FishBase (https://fishbase.org/) and the localities of samples in this study (Fig. 1), *S.maculatus* and *S.regalis* are both distributed in the western Atlantic Ocean, and *S.regalis* has a wider range. Banford et al. (1999) found little difference between the mitochondrial genomes of *S.maculatus* and *S.regalis* and hypothesized that the hybridization with *S.regalis* resulted in the introgressive loss of the *S.maculatus* mtDNA genome. Based on the 2% threshold and with reference to the geographic distributions of the two species, we speculated that *S.maculatus* and *S.regalis* are the same species. However, it is possible that introgressive hybridization affected the results, and we cannot exclude the possibility that the two are actually separate species.

The discovery of cryptic species in this study expands current estimates of biodiversity and allows better precautionary and scientifically management, which is important to plan reasonable conservation strategies (Loxdale et al. 2016). However, owing to the wide geographical distribution of the samples, it was difficult to obtain representative samples for comparison, leading to the potential for species misidentification. In addition, the maternally inherited mitochondrial COI does not reflect bi-parentally inherited nuclear genome information, making it difficult to distinguish introgressive hybridization. Therefore, future studies of species identification should combine morphometric, nuclear genetic markers, and biological analyses to further clarify the taxonomic status of the genus *Scomberomorus* so as not to destroy the available resources (Wuketits 1997).

## Supplementary materials

Supplementary material 1 DNA barcoding of *Scomberomorus* (Scombridae, Actinopterygii) reveals cryptic diversity and misidentifications Data type Data and image (word file). Explanation note Specimen information of *Scomberomorus* in this study. Test of the substitution saturation of COI gene sequences of the genus *Scomberomorus*. Results of 14 *Scomberomorus* species based on the BIN analysis. Results of 14 *Scomberomorus* species based on the K/θ method. NJ tree based on the COI sequences of 14 *Scomberomorus* species. The green clades represent five species for which the species delimitation result is different based on morphology and the NJ tree: *S.commerson*, *S.guttatus*, *S.plurilineatus*, *S.regalis*, and *S.maculatus*. MT680627 is the outgroup. Numbers near the branches are bootstrap values. ABGD analysis of the genus *Scomberomorus*. Copyright notice This dataset is made available under the Open Database License (http://opendatacommons.org/licenses/odbl/1.0/). The Open Database License (ODbL) is a license agreement intended to allow users to freely share, modify, and use this Dataset while maintaining this same freedom for others, provided that the original source and author(s) are credited. https://binary.pensoft.net/file/781948Xiao-Shu Zeng, Cheng-He Sun1, Xiao-Ying Huang, Ye-Ling Lao, Jin-Long Huang, Sha Li, Qun Zhang
