# Supplementary material for: ﻿DNA barcoding of Scomberomorus (Scombridae, Actinopterygii) reveals cryptic diversity and misidentifications
Source: Zookeys. 2022 Dec 14;1135:157–70. doi: 10.3897/zookeys.1135.93631 (PMC9836712; doi:10.3897/zookeys.1135.93631)
Supplement: Supplementary material 1 — DNA barcoding of Scomberomorus (Scombridae, Actinopterygii) reveals cryptic diversity and misidentifications [file zookeys-1135-157_article-93631__-s001.docx]

**Table S1.** Specimen information of *Scomberomorus* in this study

| Species | Sampling localities | Number | Catalog number | Sequences accession | Publication |
| --- | --- | --- | --- | --- | --- |
| *Scomberomorus plurilineatus* | South Africa | 6 | S.plu.AFR.1–6 | JF494458–JF494460  /DSLAG600-10/DSLAG1283-11/DSLAG1287-11 | Steinke et al. 2016 |
|  | Myanmar | 2 | S.plu.MYA.1–2 | MH235714/MH235715 | Direct Submission |
|  | Bangladesh | 2 | S.plu.BAN.1–2 | MH230978/MT012637 | Ahmed et al. 2021 |
|  | Singapore | 1 | S.plu.SIN.1 | MZ314746 | Neo et al. 2022 |
| *Scomberomorus munroi* | Australia | 5 | S.mun.AUS.1–6 | DQ107660–DQ107663/DQ107675 | Ward et al. 2005 |
|  |  | 1 | S.mun.AUS.6 | JX559739 | Begg et al. 2005 |
| *Scomberomorus semifasciatus* | Australia | 4 | S.sem.AUS.1–4 | DQ107654–DQ107657 | Ward et al. 2005 |
|  |  | 1 | S.sem.AUS.5 | JX559745 | Broderick et al. 2011 |
| *Scomberomorus queenslandicus* | Australia | 9 | S.que.AUS.1–9 | DQ107653/DQ107664–DQ107667 | Ward et al. 2005 |
|  |  |  |  | FOAC484-05/FOAO1817-19/FOAO1412-18/FOAH628-08 | Direct Submission |
| *Scomberomorus sp.* | Guangdong, China | 3 | S.sp.YSP.1–3 | This study | \ |
| *Scomberomorus cavalla* | United States | 1 | S.cav.USA.1 | DQ536428 | Broughton and Reneau 2006 |
|  |  | 1 | S.cav.USA.2 | KF461231 | Deeds et al. 2014 |
|  |  | 2 | S.cav.USA.3–4 | BCOLL388-08/BCOLL211-06 | Direcct submit |
|  | Mexico | 4 | S.cav.MEX.1–4 | MG837980/MG837982–MG837984 | Sarmiento-Camacho and Valdez-Moreno 2018 |
|  |  | 3 | S.cav.MEX.5–7 | HM379773/GU225660/GU225662 | Valdez-Moreno et al. 2010 |
|  | South Atlantic | 1 | S.cav.ALT.1 | MH378673 | Direct Submission |
| *Scomberomorus niphonius* | Lushi, Jiangsu, China | 4 | S.nip.SLS.1–4 | This study | \ |
|  | Yankou,Shandong, China | 3 | S.nip.LYK.1–3 | This study | \ |
|  | RaoPing,Guangdong, China | 2 | S.nip.YRP.1–2 | This study | \ |
|  | Donggang, Liaoning, China | 3 | S.nip.LDG.1–3 | This study | \ |
|  | Zhejiang, China | 2 | S.nip.ZSM.1, S.nip.ZZS.1 | KY228987/HM068273 | Direct Submission |
| *Scomberomorus guttatus* | Guangzhou, Guangdong, South China Sea, China | 2 | S.gut.YSP.1–2 | This study | \ |
|  | Yangjiang, Guangdong, South China Sea, China | 2 | S.gut.YYJ.1–2 | This study | \ |
|  | Haikou, Hainan, China | 1 | S.gut.QHK.1 | This study | \ |
|  | Lianyungang, Jiangsu, Yellow sea, China | 1 | S.gut.SLYG.1 | This study | \ |
|  | South China Sea, China | 1 | S.gut.SCS.1 | This study | \ |
|  | Bay of Bengal, Bangladesh | 5 | S.gut.BAN.1–5 | MH230970/MK988543/MK988517/MN083124/SUN060-18 | Direct Submission |
|  | the Java sea, Indonesia | 1 | S.gut.INDO.1 | MH085912 | Andriyono et al. 2020 |
|  |  | 1 | S.gut.INDO.2 | GU674020 | Direct Submission |
|  | the Arabian Sea, India | 1 | S.gut.IND.1 | MK541594 | Direct Submission |
|  | the Arabian Gulf, Saudi Arabia | 1 | S.gut.SA.1 | KU499561 | Direct Submission |
|  | South China Sea, Malaysia | 1 | S.gut.MAL.1 | BCIMS064-13 | Direct Submission |
| *Scomberomorus maculatus* | United States | 8 | S.mac.USA.1–4, 8–11 | UKFBJ681-08/BCOLL212-06/OCARH1173-12/  OCARH1179-12/MT455200/MT456078/MT455832/MH378629 | Direct Submission |
|  |  | 1 | S.mac.USA.5 | MH379061 | Stoeckle et al. 2018 |
|  |  | 2 | S.mac.USA.6–7 | KF461233/KF461232 | Deeds et al. 2014 |
|  | Mexico | 5 | S.mac.MEX.1–5 | MXV344-11/MXV345-11/HQ575755–HQ575757 | Valdez-Moreno et al. 2019 |
| *Scomberomorus regalis* | Brazil | 3 | S.reg.BRA.1–3 | JX297374/MFSP2105-12/GU702368 | Ribeiro et al. 2012 |
|  | Belize | 3 | S.reg.BEL.1–3 | JQ840989/JQ840988/JQ840682 | Weigt et al. 2012 |
|  | Trinidad and Tobago | 1 | S.reg.TAT.1 | JQ843042 | Weigt et al. 2012 |
|  | Bahamas | 1 | S.reg.BAH.1 | JQ839886 | Weigt et al. 2012 |
|  | Mexico | 1 | S.reg.MEX.1 | GU225663 | [Valdez-Moreno et al. 2010](https://doi.org/10.1071/MF09222) |
|  | United States | 3 | S.reg.USA.1–3 | HQ025013/HQ025015/HQ025016 | Handy et al. 2011 |
| *Scomberomorus brasiliensis* | Netherlands | 1 | S.bra.NET.1 | SABA071-11 | Direct Submission |
|  | Brazil | 13 | S.bra.BRA.1–3 | JX124890/JX124892/JX124893  /GU702363/GU702365/GU702366  /JQ365542/JQ365544–JQ365547/JQ365549/JQ365550 | Ribeiro et al. 2012 |
| *Scomberomorus concolor* | Mexico | 2 | S.con.MEX.1–2 | KY091265/KX925518 | Bayona-Vásquez et al. 2018 |
| *Scomberomorus sierra* | Ecuador, Galapagos, Isabela | 1 | S.sie.ECU.1 | LIDMA1247-12 | Direct Submission |
|  | Mexico | 2 | S.sie.MEX.1–2 | KX925517/HQ974552 | Bayona-Vásquez et al. 2018 |
|  | United States | 1 | S.sie.USA.1 | GU440514 | Direct Submission |
| *Scomberomorus commerson* | Dongxing, Guangxi, China | 3 | S.com.GDX.1–3 | This study | \ |
|  | Yangjiang, Guangdong, China | 3 | S.com.YYJ.1–3 | This study | \ |
|  | Rao Ping, Guangdong, China | 2 | S.com.YRP.1–2 | This study | \ |
|  | Jieshi,Guangdong, China | 4 | S.com.YJS.1–4 | This study | \ |
|  | India | 4 | S.com.IND.1–4 | ANGEN176-15/MH139918/KX227720/KM677209 | Direct Submission |
|  | Turkey | 1 | S.com.TUR.1 | KC501338 | Keskin and Atar 2013 |
|  | South Africa | 1 | S.com.AFR.1 | HM007790 | Cawthorn et al. 2011 |
|  |  | 1 | S.com.AFR.2 | JF494453 | Steinke et al. 2016 |
|  |  | 1 | S.com.AFR.3 | DQ885054 | Zemlak et al. 2009 |
|  | Philippines | 1 | S.com.PHI.1 | KF809420 | Direct Submission |
|  | United Arab Emirates | 1 | S.com.UAE.1 | MT076822 | Ludt et al. 2020 |
|  | Saudi Arabia | 1 | S.com.SA.1 | KU179067 | Direct Submission |
|  | Lebanon | 1 | S.com.LEB.1 | KR861556 | Bariche et al. 2015 |
|  | Australia | 1 | S.com.AUS.1 | FOAH592-08 | Direct Submission |

**Table S2.** Test of the substitution saturation of *COI* gene sequences of the genus *Scomberomorus*

| NumOTU | Iss | Iss.cSym | T | DF | P | Iss.cAsym | T | DF | P |
| --- | --- | --- | --- | --- | --- | --- | --- | --- | --- |
| 4 | 0.150 | 0.805 | 42.780 | 651 | 0.0000 | 0.774 | 40.748 | 651 | < 0.0001 |
| 8 | 0.151 | 0.765 | 36.742 | 651 | 0.0000 | 0.655 | 30.179 | 651 | < 0.0001 |
| 16 | 0.150 | 0.744 | 34.525 | 651 | 0.0000 | 0.534 | 22.329 | 651 | < 0.0001 |
| 32 | 0.155 | 0.718 | 31.806 | 651 | 0.0000 | 0.391 | 13.341 | 651 | < 0.0001 |

DF: the sequence length, P: the test significant value, Iss.cSym: the symmetry test, ISS.cAsym: the asymmetry test

**Table S3.** Results of 14 *Scomberomorus* species based on the BIN analysis

| OTU | Mean | Max | Count | NN Dist |
| --- | --- | --- | --- | --- |
| OTU-1 | 0.59 | 1.08 | 4 | 2.46 |
| OTU-2 | 0.31 | 0.31 | 2 | 2.46 |
| OTU-3 | 0.27 | 0.47 | 14 | 2 |
| OTU-4 | 0.31 | 1.08 | 28 | 2 |
| OTU-5 | 0 | 0 | 3 | 8.14 |
| OTU-6 | 0.06 | 0.15 | 5 | 12.14 |
| OTU-7 | 0.07 | 0.31 | 12 | 10.45 |
| OTU-8 | 0.46 | 1.08 | 14 | 8.14 |
| OTU-9 | 0.05 | 0.16 | 6 | 8.91 |
| OTU-10 | 0 | 0 | 1 | 5.72 |
| OTU-11 | 0.45 | 1.06 | 5 | 5.72 |
| OTU-12 | 0 | 0 | 6 | 5.66 |
| OTU-13 | 0 | 0 | 2 | 1.69 |
| OTU-14 | 0.26 | 0.66 | 9 | 1.69 |
| OTU-15 | 0.26 | 0.49 | 5 | 8.47 |
| OTU-16 | 0.68 | 1.38 | 15 | 3.07 |
| OTU-17 | 0.34 | 0.64 | 9 | 3.07 |
| OTU-18 | 0.25 | 0.66 | 10 | 3.91 |

**Table S4.** Results of 14 *Scomberomorus* species based on the K/θ method

| Morphological species | OTU | Clade (BI) | Clade (NJ) | K | θ | K/θ |
| --- | --- | --- | --- | --- | --- | --- |
| *S. sierra* | OTU-1 | 1 | 15 | N/A | 0.0068 | N/A |
| *S. concolor* | OTU-2 | 2 | 16 | N/A | 0.0036 | N/A |
| *S. brasiliensis* | OTU-3 | 3 | 17 | N/A | 0.0031 | N/A |
| *S. maculatus* & S. regalis | OTU-4 | 4 | 18 | N/A | 0.0031 | N/A |
| *S. sp* | OTU-5 | 5 | 4 | N/A | 0 | N/A |
| *S. semifasciatus* | OTU-6 | 6 | 13 | N/A | 0.0007 | N/A |
| *S. cavalla* | OTU-7 | 7 | 14 | N/A | 0.0003 | N/A |
| *S. niphonius* | OTU-8 | 8 | 6 | N/A | 0.0049 | N/A |
| *S. munroi* | OTU-9 | 9 | 5 | N/A | 0.0006 | N/A |
| *S. guttatus* | OTU-10 | 10 | 12 | 0.023 | 0.0026 | 8.8 |
|  | OTU-11 | 11 | 11 | 0.023 | 0.0047 | 4.9 |
|  | OTU-13 | 13 | 9 | 0.023 | 0 | N/A |
|  | OTU-14 | 14 | 10 | 0.023 | N/A | N/A |
| *S. plurilineatus* | OTU-12 | 12 | 8 | 0.103 | 0 | N/A |
|  | OTU-15 | 15 | 7 | 0.103 | 0.0028 | 36.8 |
| *S. commerson* | OTU-16 | 16 | 3 | 0.039 | 0.0075 | 5.2 |
|  | OTU-17 | 17 | 2 | 0.039 | 0.0038 | 10.3 |
| *S. queenslandicus* | OTU-18 | 18 | 1 | N/A | 0.0008 | N/A |

**
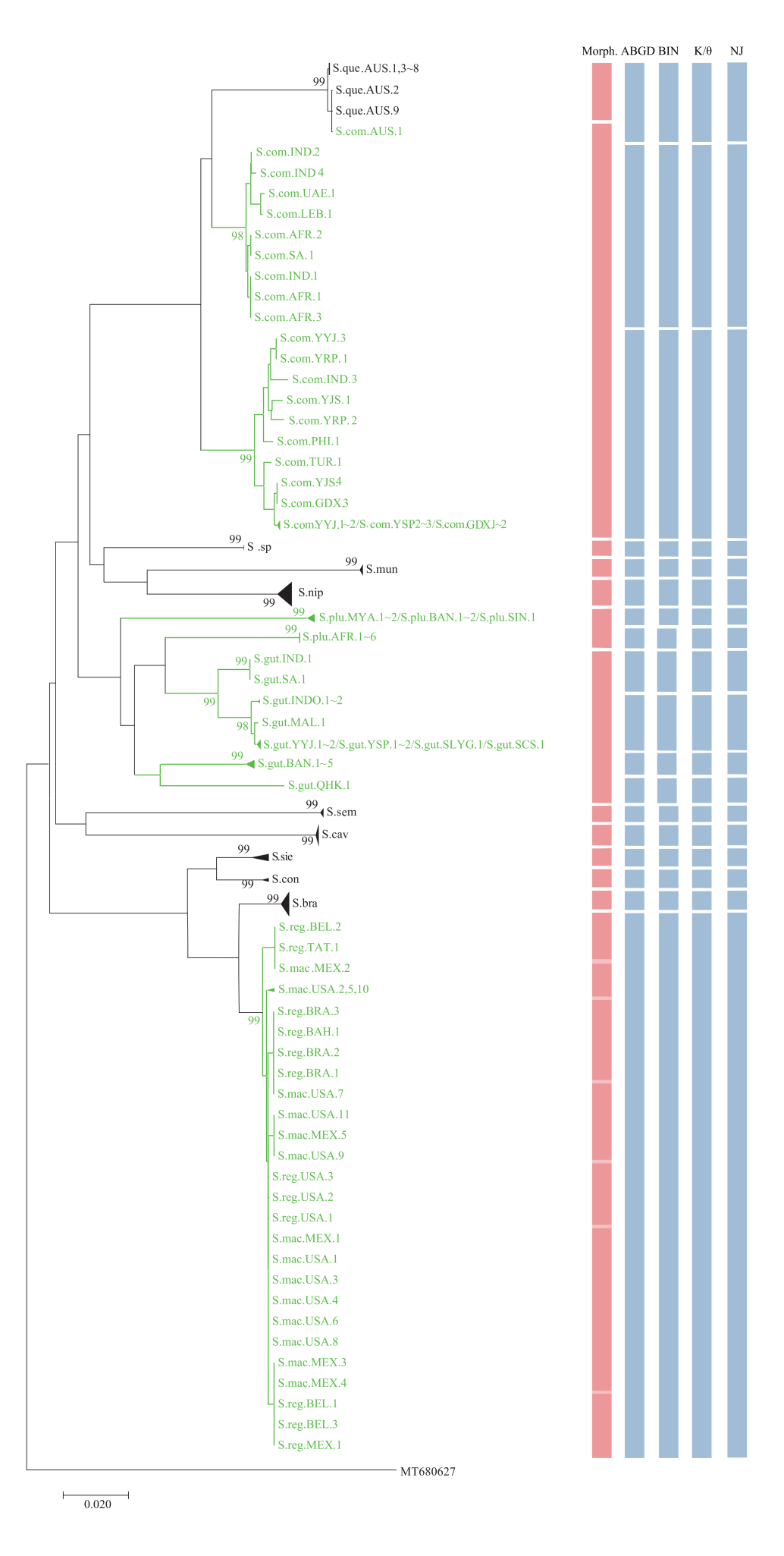
Figure S1.** NJ tree based on the *COI* sequences of 14 *Scomberomorus* species. The green clades represent five species for which the species delimitation result is different based on morphology and the NJ tree: *S. commerson*, *S. guttatus*, *S. plurilineatus*, *S. regalis*, and *S. maculatus*. MT680627 is the outgroup. Numbers near the branches are bootstrap values.


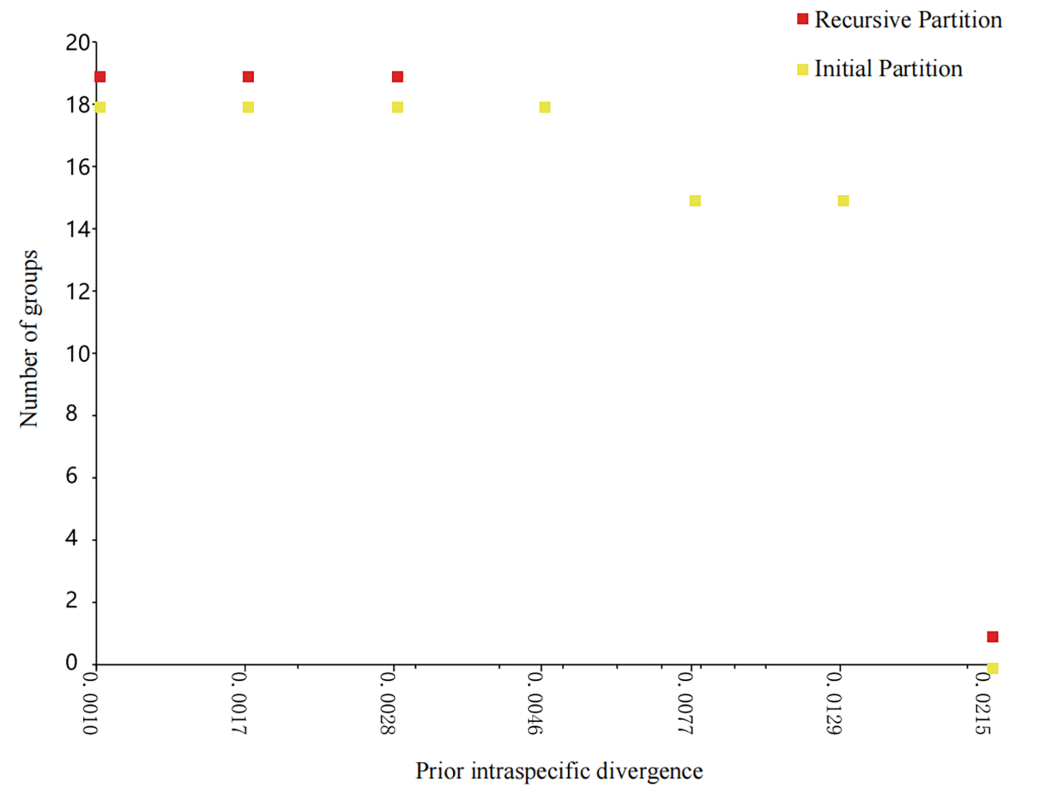


**Figure S2.** ABGD analysis of the genus *Scomberomorus*

**References**

Ahmed MS, Datta SK, Saha T, Hossain Z (2021) Molecular characterization of marine and coastal fishes of Bangladesh through DNA barcodes. Ecology and Evolution 11(9): 3696–3709. <https://doi.org/10.1002/ece3.7355>

Andriyono S, Alam M, Kim HW (2020) The Jawa and Bali Island marine fish molecular identification to improve 12S rRNA-tRNA Valin-16S rRNA partial region sequences on the GenBank database. Thalassas. International Journal of Material Science 36(2): 343–356. https://doi.org/10.1007/s41208-020-00196-x

Bariche M, Torres M, Smith C, Sayar N, Azzurro E, Baker R, Bernardi G (2015) Red Sea fishes in the Mediterranean Sea: A preliminary investigation of a biological invasion using DNA barcoding. Journal of Biogeography 42(12): 2363–2373. <https://doi.org/10.1111/jbi.12595>

Bayona-Vásquez NJ, Glenn TC, Domínguez-Domínguez O, Uribe-Alcocer M, Díaz-Jaimes P (2018) Mitochondrial genomes of the Pacific sierra mackerel Scomberomorus sierra and the Monterey Spanish mackerel Scomberomorus concolor (Perciformes, Scombridae). Conservation Genetics Resources 10(3): 471–474. <https://doi.org/10.1007/s12686-017-0851-9>

Begg GA, Bergenius MA, Cadrin SX, O’Neill MF (2005) Stock assessment of the Australian east coast spotted mackerel fishery (No. 58). CRC Reef Research Centre.

Broderick D, Ovenden JR, Buckworth RC, Newman SJ, Lester RJG, Welch DJ (2011) Genetic population structure of grey mackerel Scomberomorus semifasciatus in northern Australia. Journal of Fish Biology 79(3): 633–661. <https://doi.org/10.1111/j.1095-8649.2011.03055.x>

Broughton RE, Reneau PC (2006) Spatial covariation of mutation and nonsynonymous substitution rates in vertebrate mitochondrial genomes. Molecular Biology and Evolution 23(8): 1516–1524. <https://doi.org/10.1093/molbev/msl013>

Cawthorn DM, Steinman HA, Corli Witthuhn R (2011) Establishment of a mitochondrial DNA sequence database for the identification of fish species commercially available in South Africa. Molecular Ecology Resources 11(6): 979–991. <https://doi.org/10.1111/j.1755-0998.2011.03039.x>

Deeds JR, Handy SM, Fry F Jr, Granade H, Williams JT, Powers M, Weigt LA (2014) Protocol for building a reference standard sequence library for DNA-based seafood identification. Journal of AOAC International 97(6): 1626–1633. <https://doi.org/10.5740/jaoacint.14-111>

Handy SM, Deeds JR, Ivanova NV, Hebert PD, Hanner RH, Ormos A, Yancy HF (2011) A single-laboratory validated method for the generation of DNA barcodes for the identification of fish for regulatory compliance. Journal of AOAC International 94(1): 201–210. <https://doi.org/10.1093/jaoac/94.1.201>

Keskin E, Atar HH (2013) DNA barcoding commercially important fish species of Turkey. Molecular Ecology Resources 13(5): 788–797. <https://doi.org/10.1111/1755-0998.12120>

Ludt WB, Jabado RW, Al Hameli SM, Freeman L, Teruyama G, Chakrabarty P, Al Dhaheri SS (2020) Establishing a reference collection and DNA barcoding the coastal fishes of the United Arab Emirates. Journal of the Ocean Science Foundation 35: 54–64. https://doi.org/10.5281/zenodo.3934741

Neo S, Kibat C, Wainwright BJ (2022) Seafood mislabelling in Singapore. Food Control 135: 108821. <https://doi.org/10.1016/j.foodcont.2022.108821>

Ribeiro ADO, Caires RA, Mariguela TC, Pereira LHG, Hanner R, Oliveira C (2012) DNA barcodes identify marine fishes of São Paulo State, Brazil. Molecular Ecology Resources 12(6): 1012–1020. <https://doi.org/10.1111/1755-0998.12007>

Sarmiento-Camacho S, Valdez-Moreno M (2018) DNA barcode identification of commercial fish sold in Mexican markets. Genome 61(6): 457–466. <https://doi.org/10.1139/gen-2017-0222>

Steinke D, Connell AD, Hebert PD (2016) Linking adults and immatures of South African marine fishes. Genome 59(11): 959-967. <https://doi.org/10.1139/gen-2015-021>

Stoeckle MY, Mishu MD, Charlop-Powers Z (2018) GoFish: a streamlined environmental DNA presence/absence assay for marine vertebrates. bioRxiv 331322. <https://doi.org/10.1101/331322>

Valdez-Moreno M, Ivanova NV, Elias-Gutierrez M, Pedersen SL, Bessonov K, Hebert PD (2019) Using eDNA to biomonitor the fish community in a tropical oligotrophic lake. PLoS One 14(4): e0215505. <https://doi.org/10.1371/journal.pone.0215505>

Valdez-Moreno M, Vásquez-Yeomans L, Elías-Gutiérrez M, Ivanova NV, Hebert PD (2010) Using DNA barcodes to connect adults and early life stages of marine fishes from the Yucatan Peninsula, Mexico: Potential in fisheries management. Marine and Freshwater Research 61(6): 655–671. <https://doi.org/10.1071/MF09222>

Ward RD, Zemlak TS, Innes BH, Last PR, Hebert PDN (2005) DNA barcoding Australia’s fish species. Philosophical Transactions of the Royal Society B: Biological Sciences 360(1462): 1847–1857. <https://doi.org/10.1098/rstb.2005.1716>

Weigt LA, Baldwin CC, Driskell A, Smith DG, Ormos A, Reyier EA (2012) Using DNA barcoding to assess Caribbean reef fish biodiversity: Expanding taxonomic and geographic coverage. PLoS One 7(7): e41059. <https://doi.org/10.1371/journal.pone.0041059>

Zemlak TS, Ward RD, Connell AD, Holmes BH, Hebert PDN (2009) DNA barcoding reveals overlooked marine fishes. Molecular Ecology Resources 9: 237–242. <https://doi.org/10.1111/j.1755-0998.2009.02649.x>
